# Description of *Sergentomyia phadangensis* n. sp. (Diptera, Psychodidae) of Thailand

**DOI:** 10.1186/s13071-016-1300-4

**Published:** 2016-01-15

**Authors:** Raxsina Polseela, Jerome Depaquit, Chamnarn Apiwathnasorn

**Affiliations:** Department of Microbiology and Parasitology, Faculty of Medical Science, Naresuan University, Phitsanulok, Thailand; Center of Excellence in Medical Biotechnology, Faculty of Medical Science, Naresuan University, Phitsanulok, Thailand; Université de Reims Champagne-Ardenne, ANSES, SFR Cap Santé, EA4688 – USC «transmission vectorielle et épidémiosurveillance de maladies parasitaires (VECPAR)», 51, rue Cognacq-Jay, 51096 Reims Cedex, France; Department of Medical Entomology, Faculty of Tropical Medicine, Mahidol University, Bangkok, Thailand

**Keywords:** *Phlebotomus*, *Sergentomyia*, Thailand, Caves, Molecular biology, Systematics

## Abstract

**Background:**

Since 1996, there are emerging autochthonous cases of leishmaniasis in Thailand due to *Leishmania “siamensis”* and to *L. martiniquensis* explaining a recent interest for the sand fly fauna where *Sergentomyia gemmea* and *Se. barraudi* have been considered possible vectors in the country.

**Methods:**

Field studies were undertaken in a cave of Phitsanulok Province, Thailand. Phlebotomine sandflies have been studied morphologically and some have been processed for molecular biology (sequencing of cytB rDNA).

**Results:**

A new species of sand fly, belonging to the genus *Sergentomyia*: *Se. phadangensis* n. sp., is described. The association of the male and female is supported by the homology of the sequences of cytochrome b rDNA.

**Conclusions:**

The description of a new species in Thailand is of importance in view of the existence of autochthonous leishmaniases.

## Background

As it is an area which has long been considered to be free of leishmaniasis, there are few data related to Phlebotomine sand flies in South-Eastern Asia. However, the first autochthonous South-Eastern Asian case of leishmaniasis was discovered in Thailand in 1996, followed by a total of 13 cases due to *Leishmania “siamensis”* and, surprisingly, to *L. martiniquensis* [1], a species of the French West Indies, recently described [2]. This new epidemiological datum explains a growing interest in the sand fly fauna of Thailand. A total of 26 species of phlebotomine sand flies have been recorded in this country: one *Chinius*, three *Idiophlebotomus*, eight *Phlebotomus*, one *Grassomyia* and 13 *Sergentomyia* [3–6], excluding *Nemapalpus vietnamensis* not considered to be a sand fly [7] and some doubtful records.

During field work carried out over one year in the Phadang limestone cave, the most abundant species caught - new to Science - is described in the present paper.

## Methods

### Sampling

Field studies were undertaken between February 2010 and January 2011 in Phadang cave in Noen Maprang, Phitsanulok Province, located in the lower northern part of Thailand at 16°30'58.8"N, 100°40'00.5"E. This limestone cave, situated in a mountainous area, is found in the foothills of a mountain and consists of two asymmetrical caverns. It is estimated to be 15–80 m wide, 80–100 m long, and 1–20 m high, and lies at 108 meters a.s.l. The cave may be wet or dry depending on the season and the amount of sunlight it receives. It is accessible to travelers, but is home to many bats.

### Mounting

Phlebotomine sand flies were collected using CDC miniature light traps usually installed overnight from 6 p.m. to 6 a.m. Specimens collected were stored in 96 % ethanol. Some of them were mounted *in toto* in Hoyer’s medium and others for the application of molecular biology techniques. Regarding the latter: head, thorax and genitalia were cut off in a drop of ethanol, cleared in boiling Marc-André solution and, after dehydration, mounted on slides in Canada Balsam. The anterior part of the abdomen of each specimen was dried and stored in a vial at −20 °C, before DNA extraction.

The specimens were observed under a BX53 microscope equipped with a video camera and measured using Stream motion software (Olympus, Japan). The usual keys for the identification of the sand flies from the indo-chinese region have been consulted [8–10]. Drawings were made using a *camera lucida*. Morphological terminology is that commonly used for Old World sandflies [4, 5, 8, 10–13] and we add the corresponding nomenclature proposed for Diptera [14]. Abbreviations of generic names follow [15]. All measurements are in μm.

### Molecular analysis

We decided to sequence a fragment of cytochrome b (Cyt b) which is the most used molecular marker for Phlebotomine sand fly systematic studies [16]. Genomic DNA was extracted individually from the part of the abdomen of sand flies (5 males and 5 females) using the QIAmp DNA Mini Kit (Qiagen, Germany) following the manufacturer’s instructions, modified by crushing the sand fly tissues with a piston pellet (Treff, Switzerland), and using an elution volume of 50 to 200 μl [12].

All the mtDNA and rDNA amplifications were performed in a 50 μl volume using 5 μl of extracted DNA solution and 50 pmol of each of the primers N1N-PDR and C3B-PDR [17]. The PCR mix contained (final concentrations) 10 mM Tris HCl (pH 8.3), 1.5 mM MgCl_2_, 50 mM KCl, 0.01 % Triton X 100, 200 μM dNTP each base, and 1.25 units of 5 prime Taq polymerase (Eppendorf, Germany). The cycle begins with an initial denaturation step at 94 °C for 3 min and finishes with a final extension at 68 °C for 10 min. PCRs were done with the following temperature profile: 5 cycles with 30 sec 94 °C, 40 sec 40 °C, 1 min 68 °C and 35 cycles with 30 sec 94 °C, 30 sec 44 °C, 1 min 68 °C.

Amplicons were analyzed by electrophoresis in 1.5 % agarose gel containing ethidium bromide. Direct sequencing in both directions was performed using the primers used for DNA amplification.

The correction of sequences was done using Pregap and Gap softwares included in the Staden Package [18]. Consensus sequences were aligned by the Clustal W algorithm [19] from the BioEdit 4.8.10 sequence editor [20].

## Results

### Molecular analysis

The sequences analysed in the present study have been deposited in Genbank under numbers KT266691 to KT266700. All of them are similar (100 % homology).

### Description of *Sergentomyia phadangensis* n. sp. Polseela, Depaquit & Apiwathnasorn

Genus *Sergentomyia* França & Parrot, 1920.

Species *Sergentomyia phadangensis* n. sp. Polseela, Depaquit & Apiwathnasorn.

### Female (Figs. 1 and 2, Table 1)

**Fig. 1:**
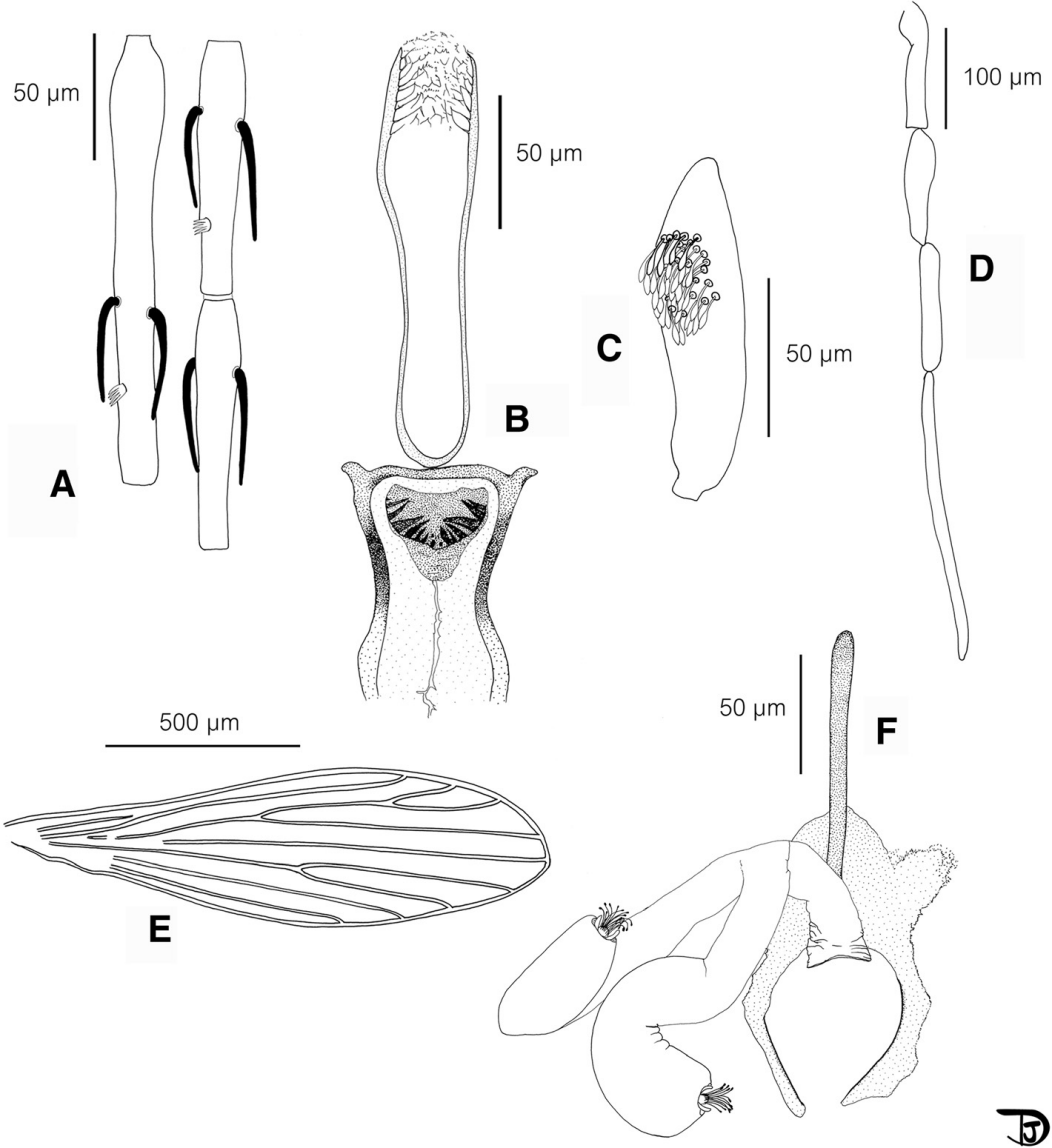
Se. *phadangensis* n. sp. female. **a** antennal segments III to V; **b** pharynx and cibarium ; **c** third palpal segment ; **d** palp; **e** wing; **f** furca and spermathecae

**Fig. 2:**
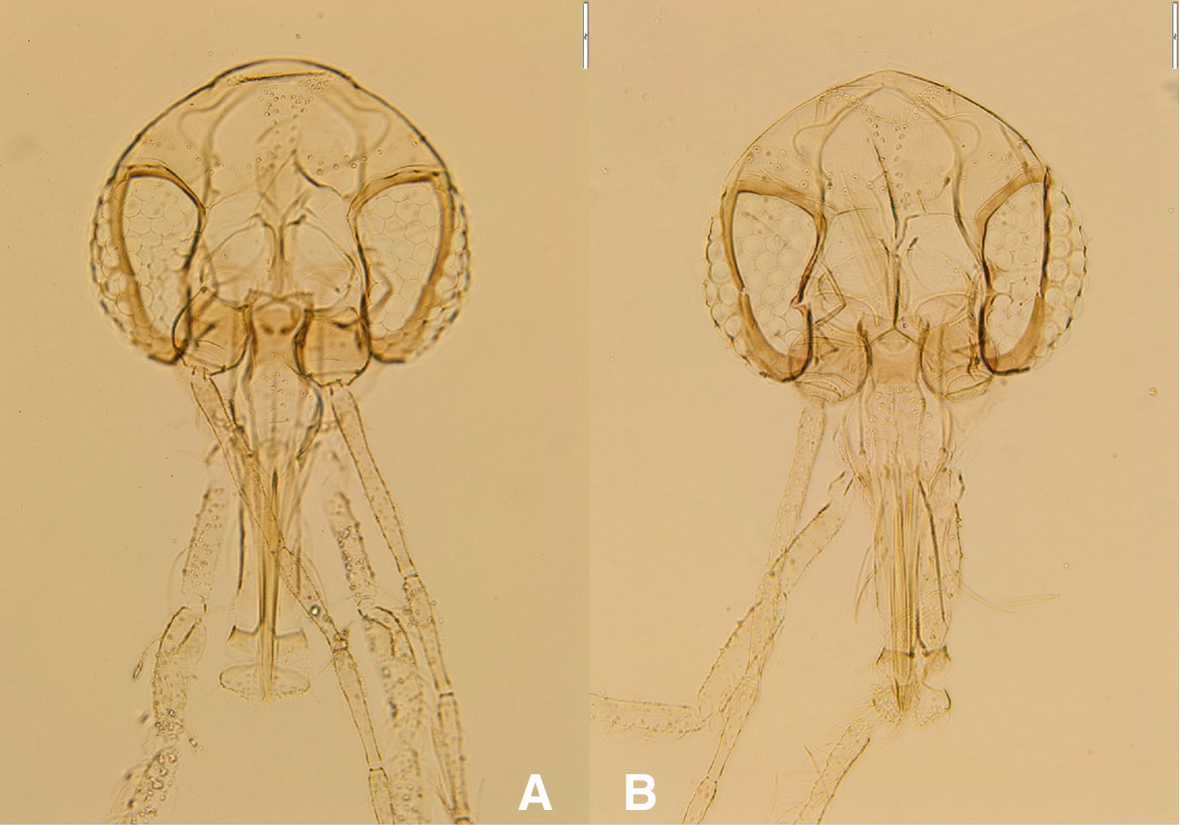
Heads of the female holotype (**a**) and of an allotype (THA39) (**b**)

**Table 1:** *Sergentomyia phadangensis* n. sp. females measurements (*n* = 16) in μm

	Holotype	Minimum	Maximum	Mean	Standard error
Head length	494	487	552	510.19	18.04
Head width	292	290	335	307.54	14.29
Eye length	155	143	188	158.69	12.55
Interocular distance	132	112	155	135.92	13.07
Clypeux length	92	86	111	97.43	8.86
AIII (= flagellomere I, F1)	165.29	140.71	165.29	158.71	8.36
AIV (= flagellomere II)	98.43	82.88	98.43	90.80	4.83
AV (= flagellomere III)	98.54	86.89	98.54	92.83	3.73
labrum-epipharynx (LE)	188.03	172.59	201.07	185.16	8.24
AIII (=FI)/LE	0.88	0.77	0.89	0.85	0.04
Palpal segment 1 (P1)	37.35	24.72	42.57	34.57	4.75
Palpal segment 1 (P2)	94.55	84.12	99.25	91.77	4.87
Palpal segment 1 (P3)	112.75	112.75	134.73	120.08	7.48
Palpal segment 1 (P4)	121.70	107.03	132.11	120.82	8.34
Palpal segment 1 (P5)	273.26	192.98	340.00	261.50	42.44
Wing length	1537.67	1383.73	1608.35	1498.53	58.02
Wing width	460.46	400.37	492.16	447.38	27.58
Alpha	288	146.00	288.00	197.00	41.17
Gamma	276	226.00	339.00	276.82	32.73
Delta	29	18.00	66.00	43.00	14.98
Pi	389	238.00	473.00	347.18	75.09
R5	1102	1010.00	1162.00	1083.09	48.58
Mesonotum length	481	481.00	599.00	522.55	39.69

The holotype (labelled THA4), and 15 paratypes specimens labelled P1, P2, P3, P4, P5, THA6, THA8, THA9, THA11, THA12, THA13, THA14, THA15, THA16 and THA19 have been examined. The labels refer to the lab where the specimens have been mounted (P: Thailand; THA: France).

*Head

Interocular suture complete.

Pharynx narrow. Discrete armature including some little teeth oriented backward.

Cibarium with a very important curved armature consisting of 6 to 8 strong and pointed teeth on each side and a few much smaller median and central ones. All these teeth are darkly pigmented in brown. Sometimes some accessory teeth in the upper and lateral parts of the cibarium. Pigment patch: wide, less pigmented than the teeth, triangular with rounded angles.

Palpal formula: 1, 2, (3, 4), 5. About forty Newstead's scales club-like in a patch on the basal face of the third segment.

Antennal formula: 2/III-XV. Relatively long ascoids, withour spur, not reaching the next articulation. AIII shorter than AIV + AV.

AIII shorter than the labrum.

Labial furca closed.

*Thorax

No setae on the mesanepisternum (proepimeral, anepisternal or katepisternal).

No pigmentation of mesonotum, pronotum, paratergite, anepisternum, metanotum, posnotum and pleura.

*Spermathecae: smooth and wide. No limit between the body and individual duct. Presence of a common basal duct.

Furca with a long and thin anterior part.

### Male (Figs. 2 and 3, Table 2)

**Fig. 3:**
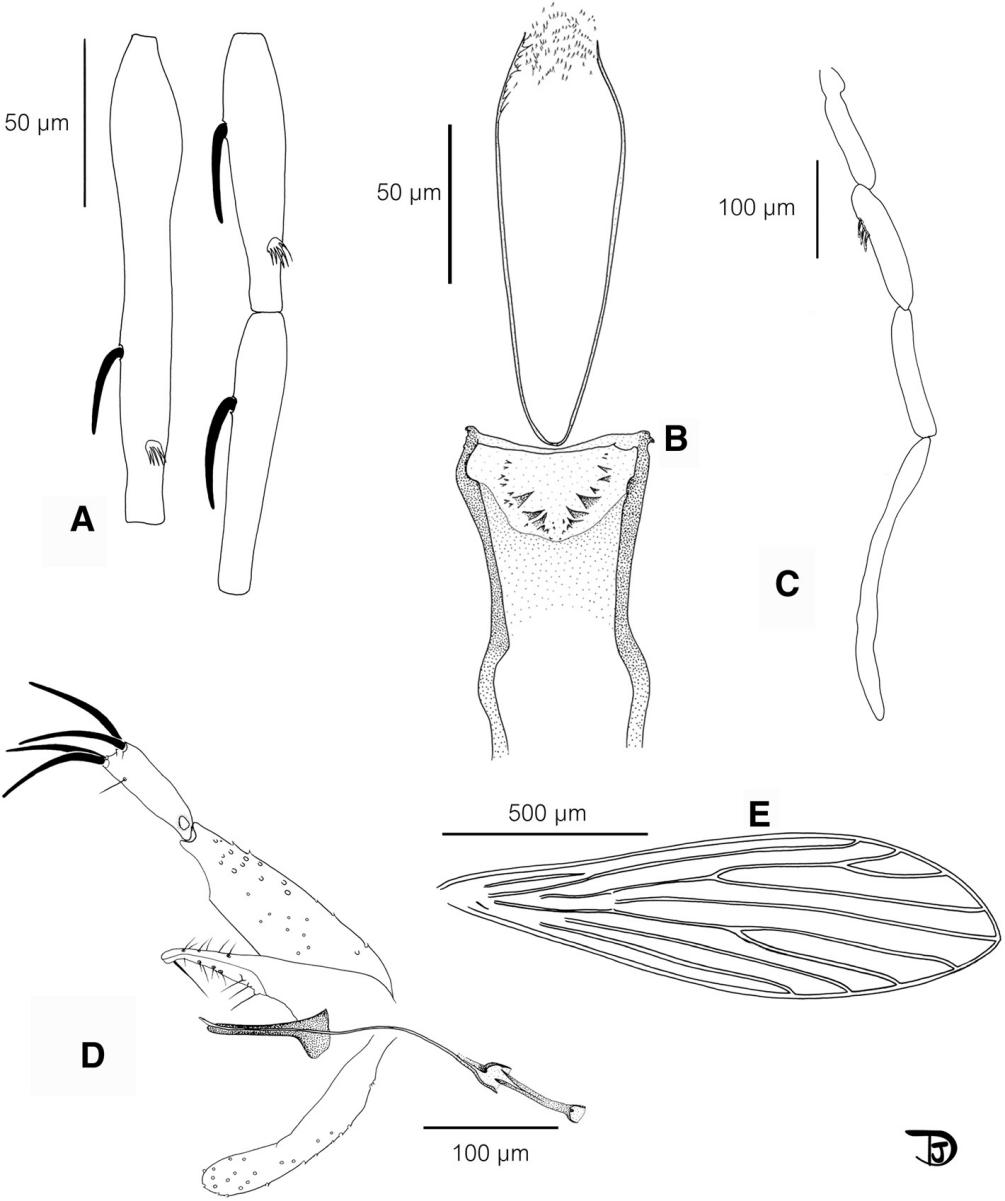
Se. *phadangensis* n. sp. male. **a** antennal segments III to V; **b** pharynx and cibarium ; **c** palp ; **d** genitalia; **e** wing

**Table 2:** *Sergentomyia phadangensis* n. sp. males measurements (*n* = 10), in μm

	Minimum	Maximum	Mean	Standard error
Head length	460	512	488.13	18.29
Head width	280	337	295.00	17.65
Eye length	152	169	158.44	5.15
Interocular distance	128	142	132.88	4.70
Clypeux length	95	131	118.33	10.82
AIII (= flagellomere I, F1)	143.60	145.29	144.65	0.91
AIV (= flagellomere II)	79.88	84.33	82.06	2.23
AV (= flagellomere III)	78.80	83.08	80.94	3.02
labrum-epipharynx (LE)	138.34	179.14	158.77	12.45
AIII (=FI)/LE	0.80	0.94	0.86	0.07
Palpal segment 1 (P1)	27.18	36.87	29.88	3.42
Palpal segment 1 (P2)	76.52	93.63	84.22	5.72
Palpal segment 1 (P3)	104.29	120.74	114.32	5.24
Palpal segment 1 (P4)	104.39	131.83	115.34	8.28
Palpal segment 1 (P5)	196.00	272.25	237.28	24.04
Wing length	1220.00	1403.09	1321.71	73.36
Wing width	361.28	411.61	395.36	17.52
Alpha	24.00	174.00	102.17	54.50
Gamma	227.00	268.00	252.40	15.60
Delta	−98.00	66.00	−9.67	61.87
Pi	300.00	349.00	325.60	17.39
R5	860.00	965.00	918.60	41.36
Mesonotum length	395.00	441.00	416.75	21.82
Coxite	193.76	214.00	202.19	6.74
Style	87.76	99.38	93.51	4.44
Aedeagus	79.99	94.21	86.38	4.00
Genital pump (GP)	70.72	92.46	80.04	7.36
Genital filaments (GF)	201.35	239.99	221.03	12.16
Surstyle (=epandrium)	143.59	194.24	164.48	18.38
Coxite/surstyle	1.08	1.41	1.24	0.13
GP/GF	2.47	3.00	2.77	0.20

Nine paratypes labelled P27, THA31, THA32, THA34, THA36, THA37, THA38, THA39 and THA40 have been examined. The labels refer to the lab where the specimens have been mounted (P: Thailand; THA: France)

*Head

Inter-ocular suture complete.

Cibarium with an important curved armature consisting of three to five large pointed teeth on each side and twelve to twenty denticles along the curve and sometimes outside this line. The latter are observable using the phase contrast option of the microscope.

No trace of a pigmented area could be seen.

Pharynx quite narrow, with a discrete armature composed of small teeth oriented backward.

Palpal formula: 1, 2, (3, 4), 5. A few Newstead's scales on the third palpal segment.

Antennal formula: 1/III-XV. AIII shorter than AIV + AV.

Labial furca closed.

*Thorax

No setae on the mesanepisternum (proepimeral, anepisternal or katepisternal).

No pigmentation of mesonotum, pronotum, paratergite, anepisternum, metanotum, posnotum and pleura.

*Genital Armature

Coxite with a few internal setae not grouped in a tuft.

Style narrow with four terminal spines. The accessory spine is implanted distally (between the distal quarter and third).

Single paramere, curved, with a rounded top.

Surstyle (=epandrium) shorter than the coxite.

Aedeagus straight, finger-like.

Type-locality: Phadang cave located in the lower northern part of Thailand at 16°30'58.8"N, 100°40'00.5"E, 108 meters above sea level.

The holotype (female) and 10 paratypes (5 females and 5 males) have been deposited in the National Science Museum of Pathumthani (Thailand). Fourteen paratypes (ten females and four males) have been deposited in the Museum national d’Histoire naturelle of Paris (France).

Etymology: the name *Sergentomyia phadangensis* n. sp. is related to the Phadang cave where it has been caught.

In accordance with section 8.5 of the International Code of Zoological Nomenclature, details of the new species have been submitted to ZooBank. The life science identifier (LSID) related to record is urn:lsid:zoobank.org:pub:8920F0C8-79BD-47AA-B367-E052C38023F9. The LSID for the new name *Sergentomyia phadangensis* is: urn:lsid:zoobank.org:act:425142C7-380D-46B0-8777-33469075C4DC.

## Discussion

For a long time, the species of the genus *Sergentomyia* were little studied as they were not regarded as vectors of any human leishmaniasis agent. However, some of them have recently been presented as possible vectors of *Leishmania* responsible for human infection [21], in Thailand too [22].

*Sergentomyia* is the genus with the greatest known diversity, surpassing the number of species of all other Old World genera (*Phlebotomus*, *Idiophlebotomus*, *Chinius*, *Spelaeophlebotomus*, *Grassomyia*, *Parvidens*, *Spelaeomyia* and *Demeillonius*) taken together [23, 24]. The species of the genus *Sergentomyia* share the following characters: a mesanepisternum without setae, abdominal tergites 2–6 usually carrying all or most recumbent hairs, a usual 1/III–XV antennal formula in males and 2/III–XV in females with some exceptions, a cibarium with an armature of teeth and/or denticles more developed in females than in males (apart from exceptions), a single paramere, a style with four terminal spines (or often 2 terminal and 2 subterminal ones) and an accessory spine.

The species *Se. phadangensis* n. sp. exhibits the characters qualifying it for inclusion in the genus *Sergentomyia*.

Mainly based on the spermathecal morphology, the genus *Sergentomyia* is subdivided into eight subgenera: *Sergentomyia* França & Parrot, 1920; *Neophlebotomus* França & Parrot, 1920; *Sintonius* Nitzulescu, 1931; *Parrotomyia* Theodor, 1948; *Rondanomyia* Theodor, 1948; *Capensomyia* Davidson; *Vattieromyia*, Depaquit, Léger & Robert, 2008, and *Trouilletomyia* Depaquit, Léger & Randrianambinintsoa, 2014. Moreover, there are a lot of unclassified species within the genus *Sergentomyia* [25].

*Se. phadangensis* n. sp. exhibits wide and smooth spermathecae and can therefore be included in the subgenus *Sergentomyia.*

**Fig. 4:**
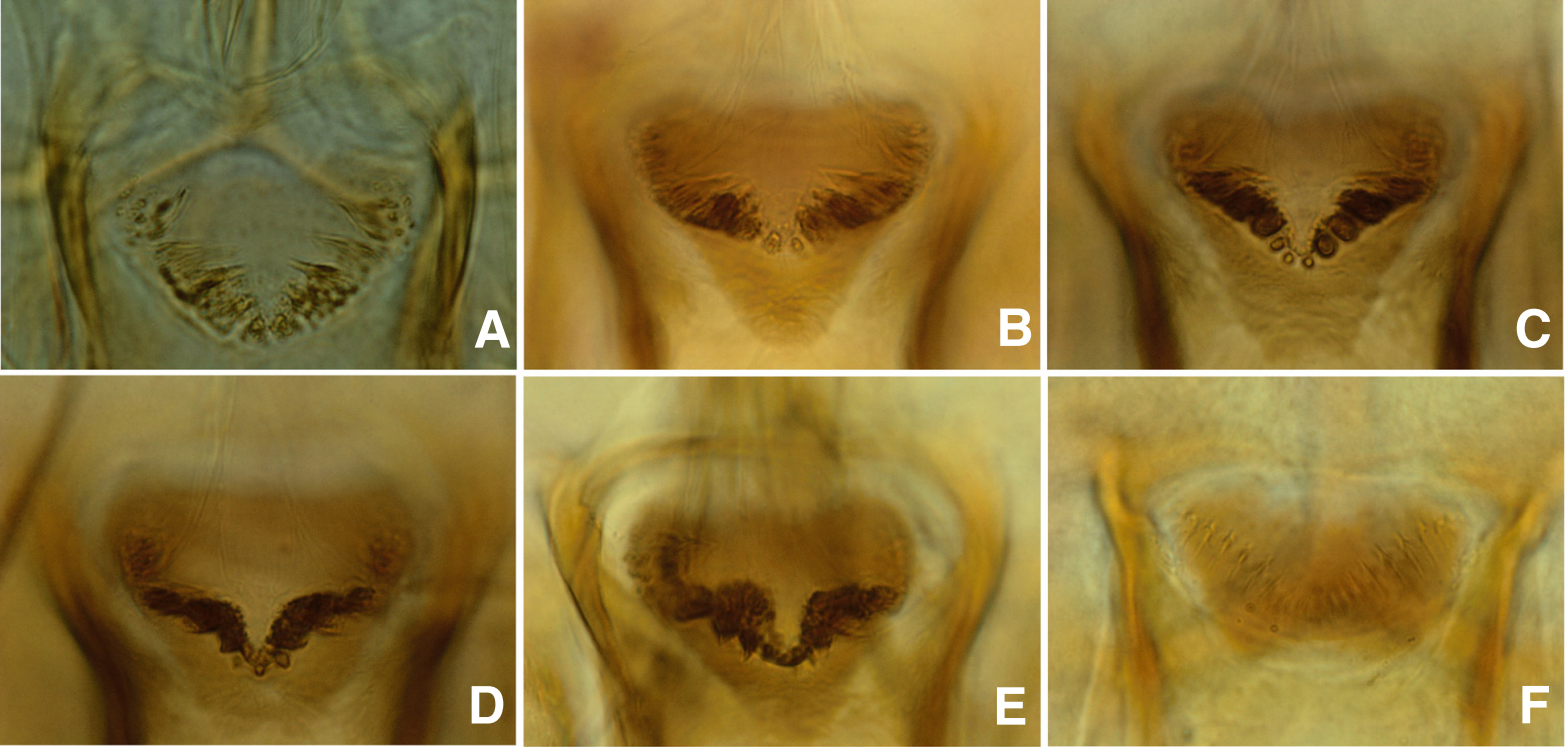
Cibariums of females of *Se. phadangensis* n. sp. (**a**, **b**, **c**, **d**, **e**) and of *Se. dentata* from Turkey (**f**)

**Fig. 5:**
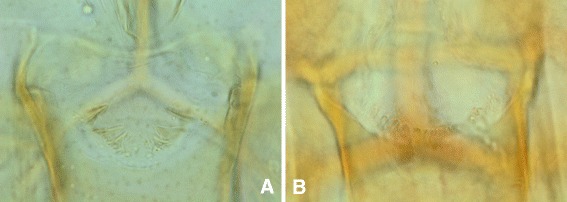
Cibariums of males of *Se. phadangensis* n. sp. (**a**) and of *Se. dentata* from Turkey (**b**)

**Fig. 6:**
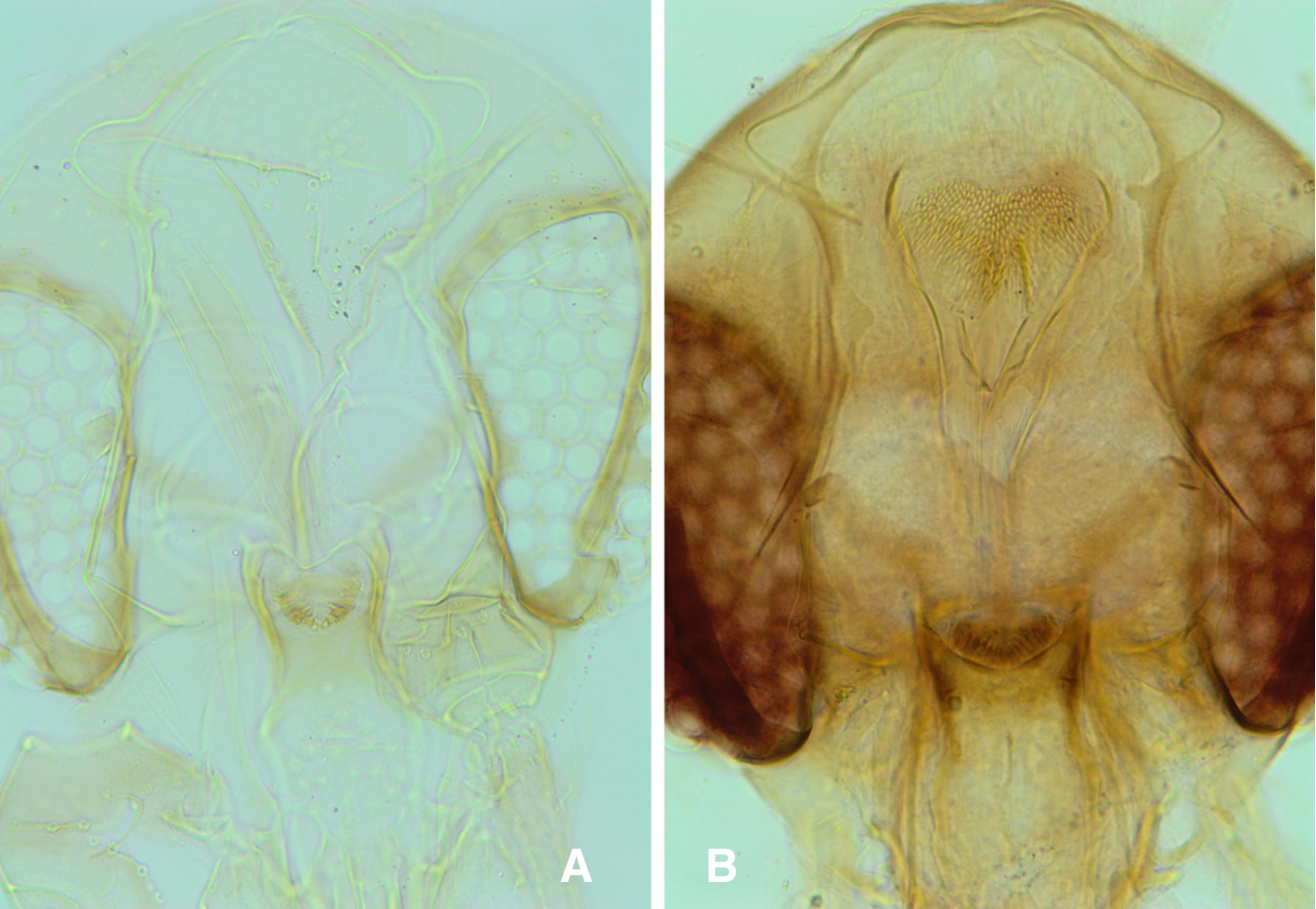
Pharynx and cibarium of males of *Se. phadangensis* n. sp. (**a**) and of *Se. dentata* from Turkey (**b**)

This new species can be easily differentiated from all the other species recorded in South-Eastern Asia by its highly developed cibarial armature in both females and males. The teeth are bigger and darker than those of the most closely related species, *Se. dentata* (Figs. 4 and 5). Moreover, the pharynx of *Se. dentata* is wide with a *Se. antennata*-like proeminent armature whereas that of *Se. phadangensis* n. sp. is narrow with a *Se. schwetzi*-like discret armature (Fig. 6). The record of *Se. dentata* in Thailand [26] should be checked in the light of the description of this new species. Initially described from Pakistan, *Se. dentata* is mainly distributed in the Middle-East and the Eastern Mediterranean basin [27].

## Conclusion

The description of a new species in Thailand is of importance taking into account the existence of autochthonous leishmaniases caused by *L. “siamensis”* and *L. martiniquensis*. The local transmission of these parasites is not explained, despite the possible role of two *Sergentomyia* species: *Se. gemmea* and *Se. barraudi*.

## Abbreviations

Cyt b: Cytochrome b
*Se*.: *Sergentomyia*
